# Dried fish dataset for Indian seafood: A machine learning application

**DOI:** 10.1016/j.dib.2024.110563

**Published:** 2024-05-29

**Authors:** Priyanka Paygude, Milind Gayakwad, Dhanashri Wategaonkar, Rajendra Pawar, Ramchandra Pujeri, Rahul Joshi

**Affiliations:** a Bharati Vidyapeeth (Deemed to be University) College of Engineering, Pune, India; b Department of Computer Engineering and Technology, Dr Vishwanath Karad, MIT World Peace University, Pune, India; c MIT School of Computing, MIT Art Design and Technology University, Pune, India; d Symbiosis Institute of Technology, Symbiosis International (Deemed University), Pune, India

**Keywords:** Dried fish classification, Dried fish dataset, Dried fish detection, Machine learning

## Abstract

Dryingfish is a simple and economical way to process the catch. It creates a profitable business for coastal communities by providing a market for their catches, even during periods of abundance. It's a traditional method to preserve fish, especially valuable in regions where fresh fish isn't readily available or affordable throughout the year. This dataset provides a rich resource of **8290 images** specifically designed for machine learning applications. It focuses on the five most popular types of dried seafood in India: prawns (shrimp), small anchovies (tingali), golden anchovies (mandeli), mackerel (bangada), and Bombay duck (bombil). To ensure high-quality data for machine learning applications for Identification and classification of different dried fish varieties, the dataset features a diverse set of images in singles and in bulk for each category. The dataset utilizes standardized lighting, background, and object pose for optimal machine learning performance. This rich dataset empowers researchers and data scientists to leverage machine learning for various applications in the Indian dried fish industry.Overall, the Dried Fish Dataset for Indian Seafood aims to leverage machine learning to improve the standardization, quality control, safety, and efficiency of the Indian dried fish industry.

Specifications Table

**Table:** 

Subject	Machine Learning, Animal Science, Food Science
Specific subject area	Dried Fish dataset with quality classification
Type of data	Images
Data collection	This dataset offers a comprehensive collection of 8290 high-quality images of dried fish captured at Bombil Market, Shukravar peth, Pune, India. It focuses on the five most popular dried seafood categories in India: Prawns (Shrimp), Small Anchovi (Tingali), Golden Anchovi (Mandeli), Mackerel (Bangada) and Bombay Duck (Bombil). For each category, images are meticulously organized into separate folders. Within each category folder, you'll find two subfolders- Single Images and Bulk Images. Single images containing close-up shots of individual fish while bulk Images showcasing multiple fish together, as typically sold in the market. We took meticulous care to capture the fish from every angle, under different lighting conditions, and with a variety of backgrounds, ensuring a rich and diverse collection. Each individual dry fish was captured under controlled lighting conditions to ensure consistent image quality. Images were saved in JPEG format and resized to a uniform resolution of 1365 × 1024 pixels for efficient storage and processing. Images were renamed sequentially for clear organization within the dataset.
Data source location	Bombil Market, Shukravar Peth, Pune, Maharashtra, India Longitude and Latitude: 18.5125° N, 73.8589° E
Data accessibility	Repository name: Dry Fish Dataset Data identification number: 10.17632/b35yrbh762.1 Direct URL to data: https://data.mendeley.com/datasets/b35yrbh762/1

## 1 Value of the Data

• This dataset [1], the first open-access collection of its kind, showcases images of five prominent Indian dry fish species.Featuring 8290 high-quality images, this dataset captures a wide range of objects categorized into five main classes, with each class containing two subcategories.
• AI models trained on this dataset will be able to automatically identify, sort, and grade various dried fish varieties according to their outward appearance. The dataset can be used to develop food recognition apps with the ability to identify dried fish dishes in images. This could be beneficial for recipe identification, dietary tracking, and automated food analysis in restaurants [[2], [3], [4]].
• This dataset can help to improve online marketplaces for dried fish by enabling image-based search for specific types and providing visual information to buyers about the appearance and condition of the product.
• Researchers can leverage the pre-trained model generated from the dry fish dataset as a starting point for their own tasks. By use of transfer learning approach, study can be a time-saving approach for tasks that require similar image recognition capabilities but might involve different objects or categories altogether.
• This dataset serves as a valuable benchmark for evaluating and comparing the performance of various computer vision algorithms in dried fish recognition tasks. This allows researchers to evaluate and improve their own models.
• This collection of images will serve as a valuable resource for researchers in food science, animal science, and various other disciplines.

## 2 Background

Drying fish is a traditional method to extend the shelf life of fish. By drying fish, the nutrients become concentrated, making it a rich source of protein and other minerals too. This is particularly valuable in regions where fresh fish isn't readily available or affordable throughout the year. Dry fish also contributes to the economic well-being of coastal communities in India. Detailed images can improve online marketplaces by enabling image-based search and providing buyers with clear visual information about the product.

There's a rising trend in applying AI and computer vision to various aspects of the food industry, including animal science, for processing, inspection, and quality control. A dry fish dataset contributes to this advancement.

## 3 Data Description

From agriculture to social sciences, image datasets are like building blocks for various fields. The “Dried Fish Dataset” aims to be a game-changer for machine learning by providing a vast and diverse collection of high-resolution images. This dataset helps develop programs that can automatically identify and classify dried fish.

To mimic real-world complexities, the dataset includes high-resolution images (1365 × 1024 pixels at 72 dpi) featuring clear and detailed visuals of dried fish. Encompassing a broad spectrum of backgrounds, lighting, and angles, the dataset features images with both single and multiple dried fish specimens. This diversity ensures model robustness in real-world scenarios.

**Fig. 1:**
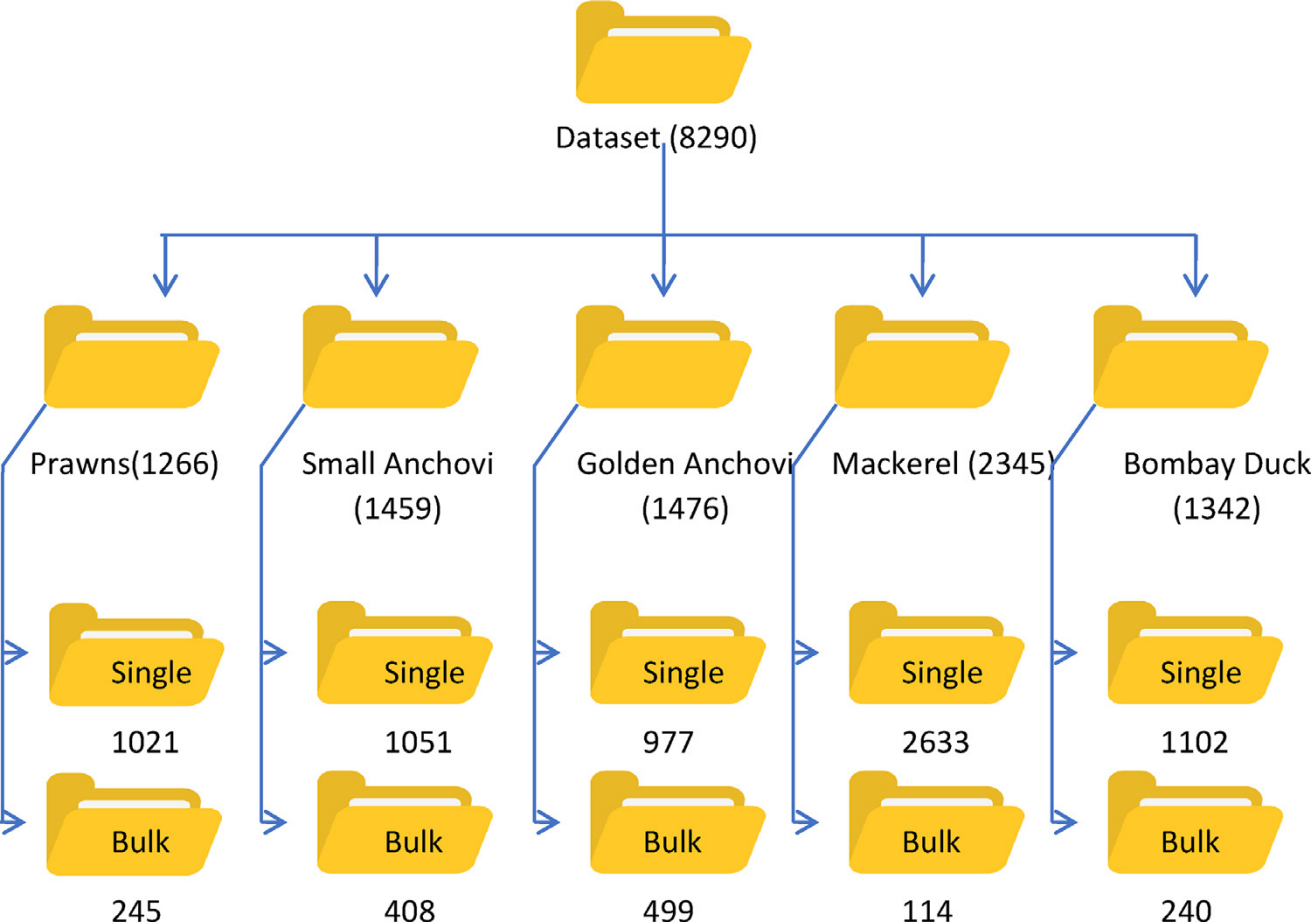
Organization of dataset.

The dataset captures the diversity of dried fish at a local market in Pune, India.It features over 8290 images across five common dried fish types: Prawns (Shrimp), Small Anchovi (Tingali), Golden Anchovi (Mandeli), Mackerel (Bangada), and Bombay Duck (Bombil). To showcase variations, each category includes both single fish and bulk quantity photos stored in separate folders.For a realistic representation, the images were captured with a consistent background but under various lighting conditions, both indoors and outdoors. Table 1 provides a detailed breakdown of image distribution across categories, and Fig. 1 visually depicts the dataset's folder structure for easy navigation.

**Table 1:** Breakdown of image distribution of the dataset.

Sr. No	Category	No. of Single Images	No. of Bulk Images	Total
1	Prawns (Shrimp)	1021	245	1266
2	Small Anchovi (Tingle)	1051	408	1459
3	Golden Anchovi (Mandeli)	977	499	1476
4	Mackerel (Bangada)	2633	114	2345
5	Bombay Duck (Bombil)	1102	240	1342
	**Total**	**6784**	**1506**	**8290**

## 4 Experimental Design, Materials and Methods

### 4.1 Experimental design

**Fig. 2:**
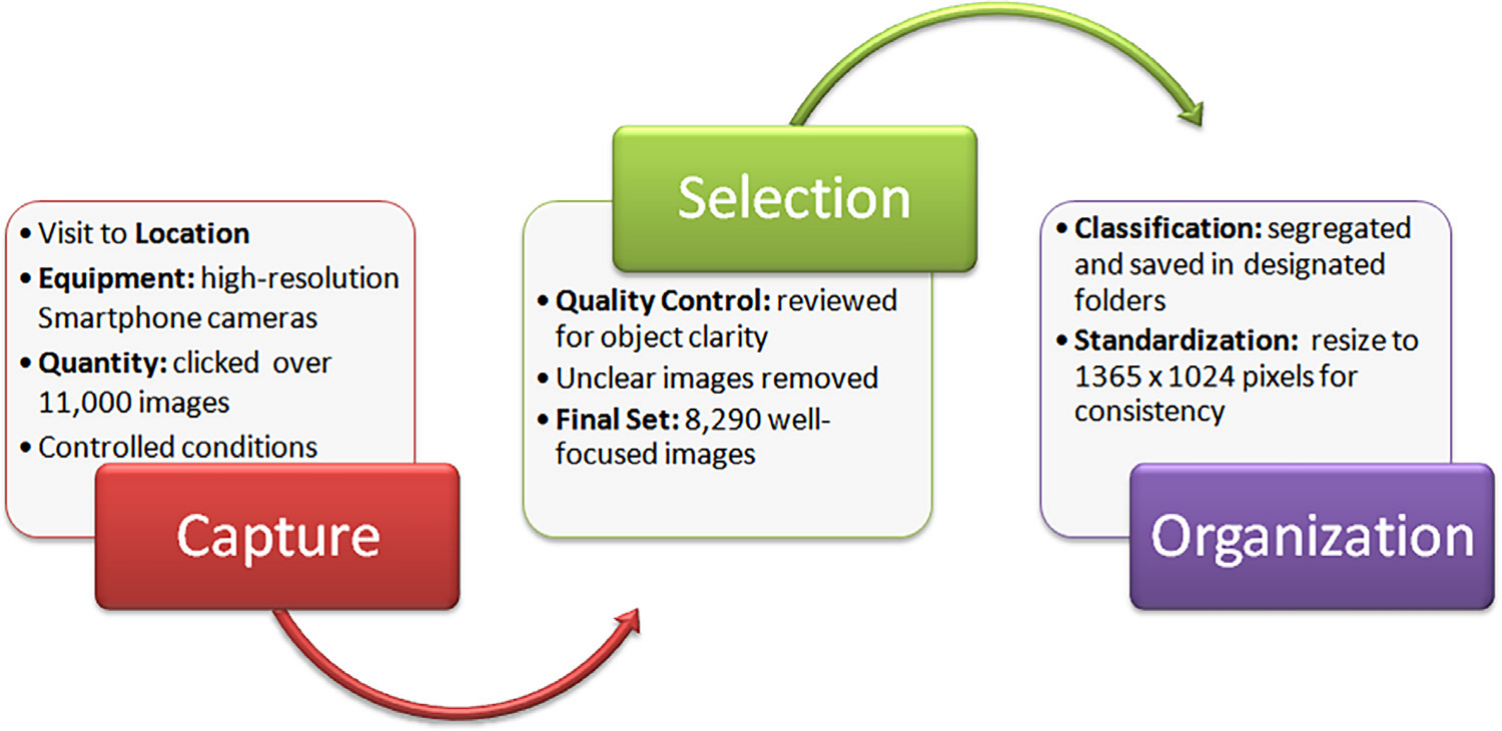
Image capturing process.

Fig. 2 illustrates the image capture process. We used two high-resolution smartphone cameras: the iPhone 6 (Apple) and the Realme 6i (Realme). Initially, over 11,000 images were captured. To ensure high quality, each image was reviewed for object clarity, and blurry images were removed during preprocessing. This resulted in a final set of 8290 well-focused images representing fish types. For efficient access, the final images were meticulously organized into designated folders based on their classification.

Dried fish were photographed under various conditions throughout September to November. This included capturing images with natural and artificial lighting, from different angles, and against same backgrounds. To ensure uniformity, a Python script was used during pre-processing to resize all images to a standard dimension of 1365 × 1024 pixels.

In essence, capturing images at the fish market formed the first step. These images then underwent a quality check and standardization process to become part of the final dataset.

Table 2 shows sample images of each category of specious in its single and bulk form.

**Table 2:** Sample Images of each category.

### 4.2 Materials or specification of image acquisition system

This section details the camera equipment used for image capture and the resulting image specifications:

1. iPhone 6 (Apple) Mobile:
  a. Make and Model: iPhone 6 (Apple) Mobile.
  b. Rear Primary Camera: 48 MP, f/2.2
2. Realme 6i:
  a. Make and Model: Realme 6i Mobile.
  b. Rear Primary Camera: 48 MP, f/1.8

**Fig. 3:**
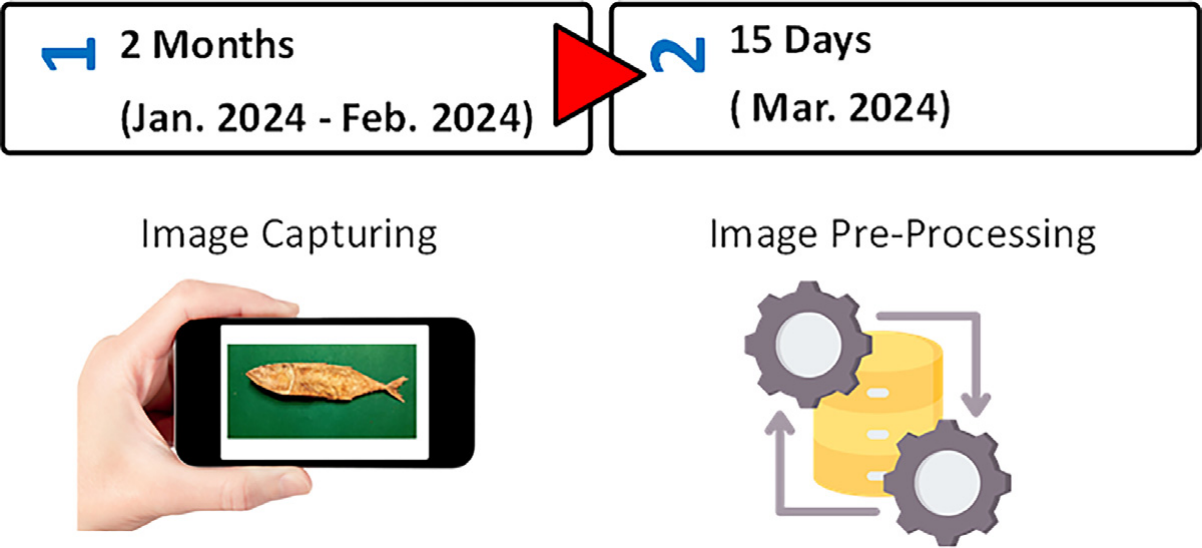
Data acquisition steps.

To guarantee consistent image quality and compatibility across the dataset, all captured images were saved in JPEG format and resized to a standard resolution of 1365 × 1024 pixels. This standardization process ensures the dataset works seamlessly with various machine learning applications (Fig. 3).

### 4.3 Method

To ensure high-quality and compatible images for our dataset, we applied a systematic pre-processing method. We used Batch Image Resizer, a popular tool known for its efficiency in batch image resizing. This allowed us to handle large image collections quickly, making it ideal for research involving image-based machine learning, image analysis, and data augmentation. After resizing, images are stored and number in sequence.

Our meticulous pre-processing goes beyond just preparing data for complex models. It sets the stage for smooth analysis throughout our research, ultimately strengthening the reliability and effectiveness of our methods.

### 4.4 Highlighting the datasetʼs value

To assess the dataset's ability to enhance machine learning model performance in dried fish classification, we conducted experiments using established pre-trained models like InceptionV3, Xception, and MobileNetV2. These models were fine-tuned on our dataset, and their accuracy in classifying dried fish types was evaluated. Table 3 evaluates Pre-trained Model Performance on Dried Fish Classification Task. This table reports the pre-training and post-training accuracy scores obtained by different pre-trained models when fine-tuned on the dried fish image dataset for the classification task [[5], [6], [7]].

**Table 3:** Comparison of ML models for pre-training and post-training on the dried fish dataset.

Machine learning model	Accuracy calculates before training on dataset	Accuracy calculates after training on dataset
Xception	40.28 %	93.89 %
InceptionV3	36.55 %	89.56 %
EfficientNetB0	42.67 %	95.23 %
ResNet50	45.56 %	94.03 %
VGG16	32.34 %	87.56 %

The dry fish dataset we created plays a key role in supercharging machine learning models like InceptionV3, Xception, EfficientNetB0, VGG16 and ResNet50. This dataset serves as a powerful training ground, allowing researchers to fine-tune these models for improved accuracy. By training AI models on this rich data, researchers can develop more reliable tools for automated sorting and grading of dried fish based solely on their appearance. This can significantly improve efficiency and consistency within the food processing industry. The dataset holds immense potential for creating food recognition applications capable of identifying dried fish dishes in images. These apps could find valuable use cases in recipe identification, dietary tracking tools, and automated food analysis in restaurants.

## Limitations

The dried or dry fish dataset lacks all categories of dried fish. It covers most popular types in Pune, India region.

## Ethics Statement

Our research aligns with Data in Brief's ethical considerations for datasets, as it does not involve animal or human subjects. Thus, confirm adherence to ethical considerations.

## CRediT authorship contribution statement

**Priyanka Paygude:** Conceptualization, Supervision, Writing – review & editing. **Milind Gayakwad:** Conceptualization, Data curation, Writing – review & editing. **Dhanashri Wategaonkar:** Conceptualization, Data curation, Writing – review & editing. **Rajendra Pawar:** Methodology. **Ramchandra Pujeri:** Methodology. **Rahul Joshi:** Writing – review & editing.

## Data Availability

Dry Fish Dataset (Original data) (Mendeley Data).

## References

[1] PAYGUDE, PRIYANKA (2024). Dry Fish Dataset”. Mendeley Data.

[2] Elliott S.A., Deleys N., Beaulaton L., Rivot E., Réveillac E., Acou A. (2023). Fisheries-dependent and-Independent data used to model the distribution of diadromous fish at-sea. Data Br..

[3] Islam M.M. (2023). Real-time dataset of pond water for fish farming using IoT devices. Data Br..

[4] Nayeem J., Dey S.K., Dey P., Himel I.A., Khatoon H. (2023). A comprehensive dataset on antimicrobial activity of indigenous Oscillatoria spp. against common bacteria causing diseases in fish and shellfish. Data Br..

[5] Thite S., Suryawanshi Y., Patil K., Chumchu P. (2024). Sugarcane leaf dataset: a dataset for disease detection and classification for machine learning applications. Data Br..

[6] Thite S., Suryawanshi Y., Patil K., Chumchu P. (2023). Coconut (Cocos nucifera) tree disease dataset: a dataset for disease detection and classification for machine learning applications. Data Br..

[7] Osisanwo F.Y., Akinsola J.E.T., Awodele O., Hinmikaiye J.O., Olakanmi O., Akinjobi J. (2017). Supervised machine learning algorithms: classification and comparison. Int. J. Comput. Trends Technol. (IJCTT).

